# Incidence of pathogen detection in blood cultures of severe sepsis and septic shock patients is higher, if blood cultures were drawn before anti-infective therapy

**DOI:** 10.1186/2197-425X-3-S1-A882

**Published:** 2015-10-01

**Authors:** CS Scheer, C Fuchs, S Rehberg, J Bast, M Vollmer, V Balau, S-O Kuhn, M Gründling

**Affiliations:** Klinik für Anästhesiologie, Universitätsmedizin Greifswald, Greifswald, Germany; Institut für Mathematik und Informatik, Ernst Moritz Arndt Universität Greifswald, Greifswald, Germany; Friedrich Loeffler Institut für Medizinische Mikrobiologie, Universitätsmedizin Greifswald, Greifswald, Germany

## Introduction

Actual guidelines for the management of severe sepsis and septic shock recommend to draw blood cultures before anti-infective therapy. The grade of recommendation for this approach is high, the evidence, however, is low (1C) [1]. Thus, data on the influence of anti-infective therapy on microbiology results in seriously ill patients are urgently warranted.

## Objectives

The objective of the present study was to describe the impact of a preexisting anti-infective therapy on the incidence of pathogen detection from blood cultures in patients with severe sepsis or septic shock.

## Methods

Therefore, we retrospectively analyzed the results of 4498 blood cultures (2 bottles, each with 8-10 ml, BD BACTEC^TM^ aerobic/anaerobic medium bottles per drawn) from 592 intensive care unit (medical and surgical) patients with severe sepsis or septic shock from 2010 to 2014 at the University Hospital of Greifswald, Germany. The results were rated in consideration of anti-infective therapy, time of origin of severe sepsis and septic shock and time of blood culture drawing. Values were calculated as percentage and 95%-confidence-intervals by Clopper-Pearson method.

## Results

**Figure 1:**
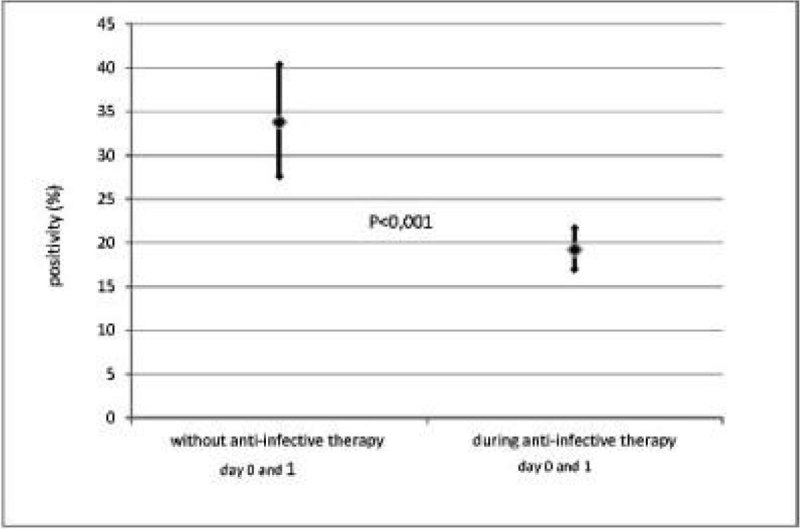
Figure 1

4210 blood cultures of 580 patients with severe sepsis or septic shock were included. 288 blood cultures of 12 patients were excluded because of incomplete data. The patients were surgical and also medical with mostly abdominal (47.6%) and pulmonary (26.4%) focus. A median of 5 (interquartile range 3-10) blood cultures per patient were drawn. Overall 20.2% (95%CI 19.0-21.5) blood cultures were positive for pathogens. 18.4% (774/4210) of blood cultures were drawn without and 81.6% (3436/4210) during anti-infective therapy. The positivity rate for all blood cultures without anti-infective therapy was 29.7% (95%CI 26.5-33.1) and 18.1% (95%CI 16.8-19.4) during anti-infective therapy (p < 0.001). Only 32.3% (1360/4210) were drawn within day 0 of origin of severe sepsis or septic shock and day 1. In this period the positivity without anti-infective therapy was 33.8% (95%CI 27.6-40.4) and 19.2% (95%CI 17.0-21.7) during anti-infective therapy (p < 0.001). Figure 1 shows the differences in positivity rate at day 0 and 1 of severe sepsis or septic shock.

## Conclusions

The present results support the current recommendation to draw blood cultures before anti-infective therapy. The highest probability of positive pathogen detection is at origin of severe sepsis or septic shock. During anti-infective the detection of pathogens is significantly decreased.
